# Returning to everyday life after discharge from a short-stay unit at the Emergency Department—a qualitative study of elderly patients’ experiences

**DOI:** 10.1080/17482631.2018.1563428

**Published:** 2019-01-29

**Authors:** Louise Moeldrup Nielsen, Lisa Gregersen Østergaard, Thomas Maribo, Hans Kirkegaard, Kirsten Schultz Petersen

**Affiliations:** a Department of Physiotherapy and Occupational Therapy, Aarhus University Hospital, Aarhus, Denmark; b Department of Occupational Therapy, VIA University College, Aarhus, Denmark; c Research Centre for Emergency Medicine, Department of Clinical Medicine, Aarhus University Hospital, Aarhus, Denmark; d Department of Public Health, Aarhus University, Aarhus, Denmark; e Centre of Research in Rehabilitation (CORIR), Department of Clinical Medicine, Aarhus University and Aarhus University Hospital, Aarhus, Denmark; f DEFACTUM, Aarhus, Denmark; g Department of Health Science and Technology, Aalborg University, Aalborg, Denmark

**Keywords:** Elderly patients, transition, rehabilitation, everyday life, discharge, daily activities, patient perspective

## Abstract

Introduction: Elderly patients often receive care and rehabilitation from different providers across healthcare settings. Collaboration between hospital and primary care providers is therefore essential to ensure that the discharge and transition of rehabilitation is coherent. However, research that focuses on elderly patients’ experiences of the discharge, and their everyday lives after, has attracted little attention. Purpose: This study explores elderly patients’ experiences of being discharged and returning to everyday lives after discharge from a short-stay unit at the Emergency Department.

Methods: Eleven qualitative interviews with elderly patients were conducted two weeks after their discharge. The transcribed interviews were analysed using systematic text condensation.

Results: The study identified four themes related to the participants experiences. In the participants perspective it was difficult, due to fatigue and pain, to perform daily activities after discharge. Participants who experienced not being prepared and clarified in relation to their discharge continued to have concerns for the future. They also experienced some challenges related to lack of being involved and lack of receiving the information needed. Conclusion: The findings contribute with impotant knowledge about elderly patients' experiences and concerns which should be taken into consideration in the discharge planning process .

## Introduction

Elderly patients are especially vulnerable when they are being discharged from hospital to home due to the complexity of their health problems (Grimmer et al., 2013; Rydeman & Tornkvist, 2006). They are at high risk of readmission, with up to 20% being readmitted within 30 days after discharge (McCabe, 2015; Salvi et al., 2007). After discharge, elderly patients need to cope with changes in their everyday lives due to their health condition as well as decreased ability to perform daily activities (Darby, Williamson, Logan, & Gladman, 2017; Jonsson, Appelros, & Fredriksson, 2017; McCabe & Kennelly, 2015, McKeown, 2007).

Elderly patients’ health problems are often more complex than those of younger patients with respect to comorbidity and limitation in performing daily activities (McCabe & Kennelly, 2015). Due to the complexity of their health problems, elderly patients typically receive care and rehabilitation from different providers across multiple healthcare settings after discharge. Effective collaboration between hospital and primary care providers is therefore essential to ensure that the discharge and transition of rehabilitation is coherent (Goncalves-Bradley, Lannin, Clemson, Cameron, & Shepperd, 2016; Hesselink, Schoonhoven, Plas, Wollersheim, & Vernooij-Dassen, 2013; Slatyer et al., 2013). A coherent discharge is characterized by collaboration between healthcare sectors with a high level of continuity and coordination, and with a clear distribution of responsibilities supported by consistent information pathways (Danish Health and Medicine Authority, 2016).

Discharging elderly patients and transitioning their rehabilitation from hospital to primary care are focal points of studies aimed at reducing risks of hospital readmission amongst elderly patients (Goncalves-Bradley et al., 2016; Karam, Radden, Berall, Cheng, & Gruneir, 2015; Lowthian, McGinnes, Brand, Barker, & Cameron, 2015). However, research that focuses on patient-perceived factors has attracted little attention. A study from 2015 revealed that frail, elderly patients experienced the transition to home as unsafe and troublesome and that their everyday lives were affected one week after discharge (Andreasen, Lund, Aadahl, & Sorensen, 2015). Another study from 2015 revealed that elderly patients experienced lack of information and participation in the discharge process (Hvalvik & Dale, 2015). Important elements in feeling prepared to come home after discharge were described by elderly patients and their relatives in a study from 2010 (Rydeman & Tornkvist, 2010). The elderly patients in that study felt prepared to come home if information and arrangements about care issues, daily activities, and contacts were organized (Rydeman & Tornkvist, 2010).

Although a number of studies have examined elderly patients’ experiences of being discharged and how they perceive their everyday lives after hospitalization, none of the identified studies were based on elderly patients’ discharge after a brief hospitalization from a short-stay unit at the Emergency Department (ED). Discharge from a short-stay unit at the ED may be especially challenging for elderly patients due to the short admission time and the short time to prepare and coordinate the discharge and the transition of rehabilitation to primary care. Due to the complexity of their health condition, which often includes comorbidity, polypharmacy, and limitations in performing daily activities, elderly patients are considered to be at a higher risk of adverse outcomes after discharge compared to younger patients (Grimmer 2013). Knowledge regarding how elderly patients experience the discharge process and how they manage their everyday life after an acute hospital stay may contribute important knowledge to inform future practice on factors of importance for the discharge and transition of rehabilitation from a short-stay unit at the ED to primary care. To our knowledge, no studies have explored elderly patients’ perspectives after discharge from a short-stay unit at the ED.

The aim of this study was therefore to explore and gain a deeper understanding of elderly patients` experiences of being discharged and their return to everyday lives after discharge from a short-stay unit the ED.

## Methods

In order to gain insight into elderly patients’ experiences of being discharged and return to everyday lives after discharge, a qualitative research design involving semi-structured interviews was chosen. Individual interviews were conducted in order to gain an understanding of elderly patients’ experiences. The study was guided by Giorgi’s methodological descriptive approach, which is based on the phenomenological philosophy of Husserl to provide an understanding of lived experiences (Giorgi, 1997, 2005; Sadala, 2002; Schiermer, 2013). The experience of being discharged and returning to everyday life is, according to the phenomenological approach, a subjective phenomenon that is experienced individually. Thus a phenomenology approach guided the study to grant access to the individual’s experience of everyday life after discharge as it is perceived by the person (Giorgi, 2005; Schiermer, 2013). According to the descriptive phenomenological approach, the lived experience should not necessarily be interpreted, but has to be described and understood in terms of how people perceive it and not from a theoretical standpoint (Bevan, 2014; Giorgi, 1997, 2005; Tuohy, Cooney, Dowling, Murphy, & Sixmith, 2013).

### Participants and study setting

The study setting was the participants’ own homes where the interviews were conducted two weeks after the participants had been discharged from a short-stay unit at the ED. We considered this an appropriate time frame for the data collection, as we expected that the participants would still be able to remember their discharge, but that they also would have experiences from their everyday life after the discharge.

The qualitative study took place alongside a quasi-experimental study that was conducted at the ED of a 1150-bed university hospital in the central region of Denmark. The purpose of the quasi-experimental study was to reduce risk of readmission by improving current discharge practice of elderly patients. Briefly, the intervention consisted of assessment of elderly patients’ performance of daily activities at the short-stay unit at the ED, referral to further rehabilitation in primary care and a follow-up at home visit the day after discharge. 144 elderly patients were allocated to receive the intervention and 231 patients to receive usual practice. A detailed description of the study is reported elsewhere (Nielsen et al., 2018).

Participants in this qualitative study were recruited among those patients who had experienced the intervention in the experimental study. The rationale for recruiting participants from the intervention group was to give the elderly patients an opportunity to express their experiences of the discharge process. The inclusion criteria were acutely admitted elderly patients over 65 years old, discharged directly to their own home from a short-stay unit at the ED and living in a larger municipality in Denmark (335,000 inhabitants). Exclusion criteria were terminal illness, severe dementia or being unable to speak and understand Danish.

Purposive sampling was adopted to ensure recruitment of participants who had experienced the discharge during the quasi-experimental study, and seeking maximum variation when it comes to diagnosis, gender, age and homecare services was applied in the sampling. This was done in order to ensure sufficient data richness which could capture some of the differences in experiences of discharge and everyday life after discharge (Creswell, 2013; Kvale & Brinkmann, 2015).

Recruitment took place from November to December 2014. Shortly before discharge, elderly patients (n = 15) were invited to participate and were given written information about the purpose of the study. Thirteen gave their consent to be contacted after discharge. Approximately one week after discharge, the patients were contacted by phone by the first author and informed about the aim of the study, that it was voluntary, and the duration of the interview. Eleven elderly patients gave their informed consent to participate: three men and eight women (Table I). Two patients out of the 13 asked chose not to participate due to their poor medical condition. Written consent to participate was obtained from the 11 participants on the day of the interviews.

**Table I.** Characteristics of the study participants (n = 11).

Participant	Gender	Age	Civil status	Reason for admission	Length of admission	Receiving home care
A	Female	83	Living alone	Back pain	2 days	+
B	Male	70	Living alone	Infection	1 day	-
C	Female	86	Living alone	Respiratory	2 days	+
D	Female	76	Living with partner	Neurological	1 day	-
E	Male	65	Living alone	Neurological	1 day	+
F	Female	83	Living alone	Infection	2 days	-
G	Female	73	Living alone	Respiratory	1 day	-
H	Female	67	Living alone	Heart problems	1 day	+
I	Female	67	Living alone	Infection	1 day	-
J	Female	76	Living with partner	Neurological	1 day	+
K	Male	76	Living with partner	Neurological	1 day	-

### Data collection

One individual interview of each of the participants, lasting 30–60 min, was conducted by the first author. In line with the phenomenological approach and the aim of the study, both open-ended and flexible questions were used to invited participants to describe their discharge, how they experienced returning to their everyday lives, and receiving rehabilitation service (Table II). The interview guide served as a guide to help stay focused on the themes, preventing unfocused interviewing but at the same time inviting participants to describe their subjective experiences as they occurred in their everyday lives after discharge. The use of open-ended questions aimed to get the participants to describe the specific situations and actions as they occurred in their everyday lives, not their general opinions. After a presentation by the interviewer and a short briefing, the participants were invited to talk freely and in detail about their experiences, and both positive and negative aspects were explored. Prompts such as “Could you give an example of this” or “Could you tell me more about that” were used to encourage the participants to tell more (Englander, 2012; Kvale & Brinkmann, 2015; Starks, 2007).

**Table II.** Interview guide.

Main theme	Questions
At the hospital	Please try to explain how you experienced being admitted to hospital.
	During your hospital stay, did anybody talk to you about how you manage different tasks at home?
Discharge	Please describe the discharge from hospital and how you experienced this.
Getting home	What was it like to come home again after hospital admission?
	What was important to you after you came home from the hospital?
	Please try to describe to me how you manage your everyday life at home.
Rehabilitation	How do you experience being offered to participate in rehabilitation?

As a phenomenological approach was used to guide both the data collection and the analysis, the first author attempted to bracket her preconception about the phenomenon to understand it as experienced by the participants (Giorgi, 2005). In order to not let the preconception influence the findings of the study, the first author wrote down her understanding of elderly patients’ discharge and discussed and reflected upon its possible implications for the data collection with the last author (Bevan, 2014). All of the interviews were audiotaped and transcribed verbatim by the first author and a research assistant.

### Data analysis

Malterud’s modification of Giorgi’s phenomenological approach to systematic text condensation was used in the analysis. This approach focuses on patterns and variations in the data, leading to a description of the participants’ experiences (Malterud, 2001, 2011, 2012). In accordance with a descriptive phenomenological methodology, an inductive approach was present in the analysis phase, as we wanted the aspects from the elderly patients’ experiences to shape the themes and codes, not theory or other preconceptions (Giorgi, 1997).

The analysis followed these steps: (1) forming an overall impression; (2) identifying and sorting meaning units; (3) condensation; and (4) synthesizing the codes into descriptions (Malterud, 2001, 2011, 2012).

Following these steps, the first author listened to all of the interviews and read the transcripts as a whole several times to gain an overview of the total content. In step 1, preliminary themes were identified and discussed with the last author. In step 2, meaning units containing information that related to the preliminary themes were identified and coded using different colours. In step 3, data were reduced and condensed to a decontextualized selection of meaning units and sorted into thematic sub-codes across the participants using a matrix. In the condensing process, the focus was on maintaining the original terminology as much as possible. Finally, the condensed meaning units were synthesized into descriptions that related to each theme and subtheme. The descriptions were discussed with the last author and four themes were agreed upon. Meaningful quotations describing the content of the subthemes were added in the description. The quotations demonstrate both similarities and differences in how the participants experienced returning to their everyday lives and how they experienced being discharged. The four main themes and subthemes are presented in Table III; they are accompanied by illustrative quotes to ensure transparency.

**Table III.** Themes and subthemes with corresponding quotes.

Theme	Subtheme	Golden quotes
Pain and fatigue limited performance of daily activities	Adapting daily activities because of fatigue	*“That’s what irritates me, I get tired too early. I am not used to that—to being tired”* (J, line 444)
	Pain that limits	*“I try to keep it (pain) down with painkillers and by walking around* *and moving and so on. But now I’ve not been moving around much because I’ve been feeling bad—it’s a vicious circle alright”* (F, lines 321–323)
	Uncertainty concerning rehabilitation	*“But it’s very overwhelming, all those things that are going to happen -* *so you’re going to have rehabilitation? yes, I am and then I don’t really* *say anything else”* (H, lines 382–383)
Frustrations and concerns	Uncertainty characterizes everyday life	*“Most of it is the psychological part—why has this happened and can it *happen again, and does it mean that I should not be alone too much? Does this mean that I should not go skiing anymore? I’ve done this alone because my wife is not a skier. But should I stop that because something could happen?”*(K, lines 184–187)*
	The time while waiting	*“It (the health condition) comes back every time, I think. Now, I hope that they will figure something out soon. The doctor was not sure and she said “before you have a diagnosis I cannot begin to treat you”, and she could *not find a diagnosis”* (F, lines 278–281)*
The importance of being involved and listened to during admission	The health professionals’	*“And he admitted me to the hospital and that was also okay. But if they had all known what they should know, right…….. and it’s often the case with doctors—A doesn’t know what B has said”* (J, lines 92–93)
	participation in medical review	*“I talked to a doctor, but it was in the evening the first day; actually, it was about 23:30 in the evening—I was simply so tired, I had been up before 6 and had slept badly the night before …. There were some things I was asked about that I really didn’t get around. I see that, I really see that today”* (G, lines 29–32)
The importance of being prepared for being discharged	Being discharged	*“Well, I did not feel so good about it (being discharged), because I was* *still in pain and all and I thought that it was a waste of time that I had* *come (to the hospital)”* (A, lines 150–151)
	Information	*“It would be nice to get something in writing. That’s always nice, so you* *can return to it. You can’t do that when it is oral. That’s what I say, you* *should be two instead of one. But if you are alone, you can get in doubt* *about what it was. It is very different if you get it in writing—then you can go back”* (G, lines 316 to 319)

Moving between the four steps was done iteratively, from the overall impression to particular parts of the transcript, identifying themes and subthemes of importance from the elderly patients’ perspective (Malterud, 2012).

### Ethical considerations

The participants in the study were elderly patients, some of which had severe disabilities. This called for special considerations in the interview situation where the interviewer tried to be attentive to the participants’ situation by trying to create a relaxed atmosphere and adjusting the length of the interview and not pressuring sensitive questions. The Ethics Committee of Central Denmark Region stated that no approval was necessary as this kind of study by Danish law does not need approval. The study was approved by the Danish Data Protection Agency (J.nr. 2012–41-0763). Basic principles for research according to the Helsinki Declaration were followed (World Medical Association, 2017). All participants were given written and oral information about the study, and written informed consent was obtained from all of the participants. Anonymity and confidentiality were secured, and participants were informed that they could withdraw from the study at any time without there being any consequences for present or future health services.

## Findings

Four themes emerged as central to the participants’ experience of return to their everyday lives and their experiences of being discharged: “pain and fatigue limited performance of daily activities”, “frustrations and concerns”, “the importance of being involved and listened to during admission”, and “the importance of being prepared for being discharged” (Table III).

### Pain and fatigue limited performance of daily activities

Since discharge, the participants experienced their everyday lives as being marked by fatigue, lack of energy, and pain, all of which limited their performance of daily activities. Fatigue and lack of energy made it more difficult to perform activities than before admission, which led to some of them feeling irritated about not being able to manage their activities. Before admission some of the participants received help to perform domestic tasks, and after their discharge some of them experienced need for either more help or receiving help more often. Sometimes, the participants were able to adapt to their changes by dividing an activity into smaller tasks that could be carried out over several days or by using assistive devices. In other cases, resting in the middle of the day could help provide the energy needed to get through the afternoon and evening.

*“That’s what irritates me, I get tired too early. I am not used to that—to being tired”* (J, line 444)

Pain was experienced as a limitation on performing daily activities. Due to pain, it was difficult for some of the participants to be as mobile as they had been previously, which meant that they did not get outside their home. Despite the fact that they considered it important to get outside, pain reduced the possibility of doing so, or made it impossible.

*“I try to keep it (pain) down with painkillers and by walking around and moving and so on. But now I’ve not been moving around much because I’ve been feeling bad—it’s a vicious circle alright”* (F, lines 321–323)

Participants’ expectations for rehabilitation after discharge were mostly positive as they expected to be able to perform daily activities as they used to before they were admitted to the ED. For participants not receiving a clear diagnosis while being admitted, thoughts of further rehabilitation were difficult to manage. Participants were concerned about their health situation and how they should handle further examinations. The uncertainty they experienced about how rehabilitation should be organized was related to whether they could continue with the specific rehabilitation training they received prior to admission and for how long. Being able to receive rehabilitation in one’s own home was considered important. Some participants stated that it could be difficult to leave home, particularly in the mornings where tiredness was prominent. It could also feel overwhelming if participants had to relate to a new therapist.

*“But it’s very overwhelming, all those things that are going to happen—so you’re going to have rehabilitation? Yes, I am and then I don’t really say anything else”* (H, lines 382–383)

Participants experienced that their everyday lives had changed after discharge, and that fatigue and pain affected their performance of daily activities, making adaptation necessary.

### Frustrations and concerns

Participants experienced frustration and concerns after being discharged. Lack of clarification as to what led to their admission created concerns that affected their everyday lives. Concerns about what caused the admission meant that some speculated on how their lives would look in the long term. Participants were also concerned about whether they would be able to perform their daily activities in the long term but at the same time, performing small daily tasks, like hanging up the laundry, could feel unimportant for some. Participant K, a 76-year-old man, was concerned about whether he and his wife would be able to travel abroad as they have done previously.

*“Most of it is the psychological part—why has this happened and can it happen again, and does it mean that I should not be alone too much? Does this mean that I should not go skiing anymore? I’ve done this alone because my wife is not a skier. But should I stop that because something could happen?”*(K, lines 184–187)

The time spent waiting for further examinations was experienced as long and difficult with many concerns such as experiencing the condition getting worse and having less energy. Participant F explained that in her case an actual treatment could not begin until there was clarity about the diagnosis.

*“It (the health condition) comes back every time, I think. Now, I hope that they will figure something out soon. The doctor was not sure and she said ‘before you have a diagnosis I cannot begin to treat you’, and she could not find a diagnosis”* (F, lines 278–281)

Some of the participants also expressed confusion about lack of clear information about which further examinations that were planned. One of the participants experienced that he did not receive the help he needed and asked for. Another participant also experienced difficulties, but related it to several different health professionals involved in her situation.

### The importance of being involved and listened to during admission

Overall, the participants experienced being treated well during admission, but they stressed that there was a hectic atmosphere with a high turnover of health professionals. Participants emphasized that it was important to be involved in decision-making and be listened to during admission, especially during the medical interview. Some experienced that the various doctors did not know what each other had said or done. Furthermore, there was waiting time to see the doctors, and it was hard to establish what was going on because the health professionals had different views related to the patients’ situation.

*“And he admitted me to the hospital and that was also okay. But if they had all known what they should know, right … and it’s often the case with doctors—A doesn’t know what B has said”* (J, lines 92–93)

The experience of not being listened to could make participants feel uninvolved in the discharge process. They found that although some physicians were good at informing about further actions and examinations, they were sometimes too busy to ask about how the participants thought and felt about the situation. Sometimes medical reviews took place late at night, which meant that the patient was too tired to ask questions about their condition or situation. This could lead to several unresolved issues in relation to discharge.

*“I talked to a doctor, but it was in the evening the first day; actually, it was about 23:30 in the evening—I was simply so tired, I had been up before 6 and had slept badly the night before …. There were some things I was asked about that I really didn’t get around. I see that, I really see that today”* (G, lines 29–32)

Some of the participants experienced waiting time during admission and that it was difficult to find out what was going on. One of the participants experienced lack of information from the healthcare staff, as they had packed her personal stuff together before she knew she was being discharged.

### The importance of being prepared for being discharged

Participants found that it was important to be prepared before discharge. They were all discharged 1–2 days after admission, which for several of them came as a surprise as they did not feel ready. Not everyone agreed with the decision about being discharged, and some felt that they had not been involved in the decision. It felt like a waste of time to be admitted when the condition did not change for the better, and they might as well have been at home in their familiar environment. Participant A, an 83-year-old woman, did not understand why she was being discharged when she was still in pain.

*“Well, I did not feel so good about it (being discharged), because I was still in pain and all and I thought that it was a waste of time that I had come (to the hospital)”* (A, lines 150–151)

Central to the participants’ experiences of a positive discharge was feeling secure about returning home. Participants who lived with a spouse experienced a sense of security, as there was someone to care of them after discharge.

Information about what was going to happen was deemed important by participants in relation to the quality of their discharge. For instance, were there going to be further examinations, rehabilitation, or care? Some of the participants experienced that they got the verbal and written information they needed in relation to their discharge, while others experienced they did not and expressed a wish for more relevant information. One of the informants experienced that lack of information about her medical treatment was problematic, because the general practitioner was not aware that she had changed medication. Furthermore, the written information could be difficult to relate to, as it was difficult to understand, and the material needed further clarification.

*“It would be nice to get something in writing. That’s always nice, so you can return to it. You can’t do that when it is oral. That’s what I say; you should be two instead of one. But if you are alone, you can get in doubt about what it was. It is very different—if you get it in writing, then you can go back”* (G, lines 316 to 319)

## Discussion

With regard to being discharged from a short-stay unit at the ED, different factors, revealed in four themes, were considered by the participants to be important for their everyday lives after discharge. Fatigue and pain affected participants’ performance of daily activities, 14 days after their discharge. Although it is possible to let caregivers take over some of the participants’ daily activities, such as housekeeping tasks, the participants expressed dissatisfaction with not being able to perform these activities themselves. Some explained how they were able to handle their decreased ability to perform daily activities by using adaptation strategies, such as dividing activities into smaller tasks, while others abstained from performing these activities. Using different strategies to adapt to new situations is well known from other studies (Aberg, Sidenvall, Hepworth, O’Reilly, & Lithell, 2005; Neiterman, Wodchis, & Bourgeault, 2015; Zakrajsek, Schuster, Guenther, & Lorentz, 2013). A study from 2005 revealed that elderly patients adopt different adaptation strategies in their everyday lives to avoid negative experiences due to their health condition (Aberg et al., 2005). The ability to continue with daily activities that are meaningful in their everyday lives is important, and different strategies can be used to reduce the gap between decreased capacity and the demands of the environment (Kielhofner, 2008). The participants in our study were in an ongoing process of learning to use adaptation strategies to perform daily activities and changing roles, habits, and routines, although some of them were challenged in that process. Their expectations for rehabilitation after discharge were mostly positive as they expected to be able to perform daily activities as they used to before they were admitted to the short-stay unit at the ED. This is in line with other studies which suggest that elderly patients admitted for acute illness should be targeted for rehabilitative services after discharge (Boyd et al., 2008; Goncalves-Bradley et al., 2016)

The participants’ everyday lives were also influenced by thoughts about their health condition as well as concerns for the future. Participants who experienced not being prepared or who did not have their diagnosis clarified felt frustrated and had concerns about whether they would be able to perform daily activities in the long term or if they would experience loss of activities. Uncertainty about their health condition and whether they should avoid performing daily activities may lead to a vicious spiral. If elderly patients avoid performing daily activities because of uncertainty, this may, in turn, decrease their ability to perform daily activities (Larsson, Ekvall Hansson, Sundquist, & Jakobsson, 2016; Mackichan, Adamson, & Gooberman-Hill, 2013).

Our findings about elderly patients’ concerns are in accordance with the findings of another study where elderly patients were concerned about how to handle their life situation after being discharged (Rydeman & Tornkvist, 2010). Our study, however, especially showed that participants who did not have a clear diagnosis felt unprepared to return home, and were concerned about the impact of their health condition on their everyday lives. It is important to take these new findings into consideration in clinical practice, and doing so may lead to the development of improved informational material about what is going to happen in relation to clarifying further the elderly patients’ condition. Moreover, provision of clear information about whether there are restrictions on certain activities may contribute to preventing decreased ability to performing daily activities and should be an important element in the discharge process.

The results of the present study show that elderly patients want to be involved and participate in their discharge from the ED, although lack of verbal and written information and lack of involvement in the discharge process were experienced by some of the participants. Not everyone agreed with the doctors’ decision about the time of discharge, and some did not feel that they had been involved in the decision. Other studies support this finding (Hvalvik & Dale, 2015). In a study from 2015, participants reported feeling invisible in the discharge process due to lack of involvement and communication with health professionals (Hvalvik & Dale, 2015). Providing sufficient information about further clarification, treatment, and rehabilitation and allowing patients to describe their perceived health challenges are necessary to involve elderly patients in decision-making and for truly informed choice (Dyrstad, Testad, & Storm, 2015).

### Methodological considerations

A strength of this study is that the qualitative individual interviews were conducted in a natural setting (e.g., the participants own home) to explore how elderly patients experiences returning to their everyday lives after discharge from a short-stay unit at the ED and how they experience being discharged. The home environment can facilitate more comfortable relationships between the researcher and participant and may encourage the participants to talk more freely during the interview (Sivell et al., 2015).

The use of a phenomenological methodological approach for guiding both the collection and analysis of data was suitable for examining patients’ experiences of being discharged and returning to their everyday lives, although it posed challenges in relation to the participants’ way of expressing themselves. The participants’ ability to express themselves and describe their experiences varied. Some of the participants were experiencing fatigue during interviews, which had an impact on the duration of the interview and their ability to concentrate. During the interviews great effort was devoted to give the participants the possibility to express their perceptions by using open-ended questions and prompts. A broad variation of participants, e.g., gender, age, and diagnosis, was included to provide nuanced insights into the elderly patients’ experiences and extensive interviews were conducted. The participants provided rich and varied data to a degree that ensured a qualified answer to the aim of the study.

When using a phenomenological methodological approach, researchers face the challenge of not letting preconceptions influence the interviewing or the findings. The researcher conducted interviews and analysis with as few preconceptions as possible. The first author primarily carried out the interviews and the analysis, and was supervised by the last author. Preconceptions were continuously reflected upon in order to achieve trustworthy findings and ensure transparency. Before conducting the interviews, the researchers had a preconception that the transition between hospital and primary care would be challenging for the participants. However, none of the participants expressed opinions on the transition; rather, they described the challenges of returning to their everyday lives. The use of a phenomenological approach allowed the researcher to pursue what was important for the participants, rather than seeking answers to predefined questions.

The use of systematic text condensation as a method for analysing data was found highly relevant as the method helped the researchers to stay focused on the participants’ experiences without the use of theory. This resulted in descriptions that provide insights into the elderly patients’ experiences of returning to their everyday lives and how they experience being discharged. These insights may help to improve the quality of discharge and transition from hospital to primary care for elderly patients in the future.

A limitation in this study was that it was conducted in a single hospital with recruitment of participants who had been involved in a clinical trial and received a specific intervention. Accordingly, we have provided a detailed description of the setting and the intervention to enhance the transferability of findings to other similar settings. Voluntary participation might also have yielded a sample that held strong views about the discharge process, which may affect the transferability of the findings. The results cannot be transferred to all elderly patients, but can be seen as illuminating patterns that can be used for hypothesis generation in further studies.

## Conclusion

Our study revealed that factors such as decreased ability to perform daily activities, not having their diagnosis clarified, and not being prepared and involved in the discharge process were important for the elderly patients’ discharge from the ED and their everyday lives after discharge. These findings contribute important knowledge about elderly patients’ experiences and concerns, which should be taken into consideration by health professionals who plan the discharge process and refer patients to further rehabilitation. Further research is needed to examine how the process of discharge from an acute setting may be improved and how focus on elderly patients’ everyday lives and their performance of daily activities can be included both at hospital and in primary care.

## Implications

Based on our findings, implication for clinical practice would emphasize the importance of qualifying how we inform and involve elderly patients in decisions related to their discharge process. Our results indicate that elderly patients whose diagnosis has not been clarified during admission experience concerns about their health condition, and that this may influence their everyday life after discharge. In addition, our findings revealed that elderly patients’ everyday lives were marked by pain and fatigue, which limited performance of daily activities. This implies that health professionals who are involved in the discharge process need to provide sufficient verbal and written information about the further process, and how it may affect the patients’ everyday life after discharge. Providing sufficient information about the patient’s health condition and its possible influence on everyday life, as well as information about further examination, treatment, and rehabilitation, may increase the patients acceptance of the situation and may lead to patients feeling prepared for discharge.
